# Fostering physical activity-related health competence after bariatric surgery with a multimodal exercise programme: A randomised controlled trial

**DOI:** 10.1007/s10865-023-00398-7

**Published:** 2023-03-02

**Authors:** Julia Schmid, Nina Schorno, André Groux, Daniel Giachino, Jörg Zehetner, Philip Nett, Christos T Nakas, David Herzig, Lia Bally

**Affiliations:** 1 Institute of Sport Science, University of Bern, Bern, Switzerland; 2 Department of Diabetes, Endocrinology, Nutritional Medicine and Metabolism UDEM, Inselspital, Bern University Hospital, University of Bern, Bern, Switzerland; 3 Department of Visceral Surgery, Lindenhofspital, Bern, Switzerland; 4 Department of Visceral Surgery, Hirslanden Klinik Beau-Site, Bern, Switzerland; 5 Department of Visceral Surgery and Medicine, Bern University Hospital, Inselspital, University of Bern, Bern, Switzerland; 6 Laboratory of Biometry, School of Agriculture, Bern University Hospital, University of Thessaly, Nea Ionia Magnesia, University Institute of Clinical Chemistry, Inselspital, University of Bern, Bern, Switzerland

**Keywords:** Health literacy, Physical literacy, Competence, Physical activity promotion, Obesity therapy, Bariatric surgery

## Abstract

Regular physical activity (PA) supports the long-term success of bariatric surgery. However, integrating health-enhancing physical activity in daily life requires specific competences. In this study, we evaluated a multimodal exercise programme to build these competences.

Forty adults who underwent bariatric surgery were randomised to a multimodal exercise programme or control group. Primary outcomes were the facets of PA-related health competences, namely the control competence for physical training, PA-specific affect regulation, motivational competence and PA-specific self-control. Secondary outcomes were PA behaviour and subjective vitality. Outcomes were assessed before, directly after the intervention and at 3 months follow-up.

Significant treatment effects were found for control competence for physical training and PA-specific self-control but not for PA-specific affect regulation and motivational competence. Significant treatment effects were further observed for self-reported exercise and subjective vitality, all in favour of the intervention group. In contrast, no treatment effect was found for device-based PA. Overall, this study provides a foundation for future research to optimise long-term post bariatric surgery outcomes.

**Supplementary Information:**

The online version contains supplementary material available at 10.1007/s10865-023-00398-7.

## Background

Bariatric surgery is an effective obesity treatment achieving substantial body weight reduction and improvement of obesity-associated comorbidities (Mingrone et al., 2015; Peterli et al., 2018; Schauer et al., 2017). However, there is increasing evidence that over the longer term, many patients experience weight regain (Sjöström, 2013). Due to the well-established positive effects of physical activity (PA) on weight management, cardiovascular risk, bone health, and wellbeing, PA support should be an integral part of follow-up care packages after bariatric surgery (Josbeno et al., 2010). However, implementation of PA support in clinical practice is still limited (James et al., 2022). Many patients do not increase their PA level after surgery and remain overly sedentary (Barbosa et al., 2019; Herring et al., 2016). Thus, there is a need for theory-based interventions, that clinicians can use to help patients to change their PA behaviour.

The physical activity-related health competence (PAHCO) model offers a theoretical base for developing PA interventions (Sudeck & Pfeifer, 2016). It describes a specific set of competences required to initiate and maintain a healthy, active lifestyle. The PAHCO model can be conceptually located at the interface between health literacy (Sørensen et al., 2012) and physical literacy (Edwards et al., 2017; Giblin et al., 2014). The PAHCO model seems particularly appropriate for the current study for at least two reasons: Firstly, because of its PA-specifity. It includes PA-typical constructs, which are often not represented adequately in more general models of health behaviour (e.g., the Health Action Process Approach; Schwarzer, 2012). Secondly, because of its interdisciplinarity. Cognitive, motivational-emotional, and physical aspects are considered to explain not only PA, but also health and wellbeing.

**Fig. 1:**
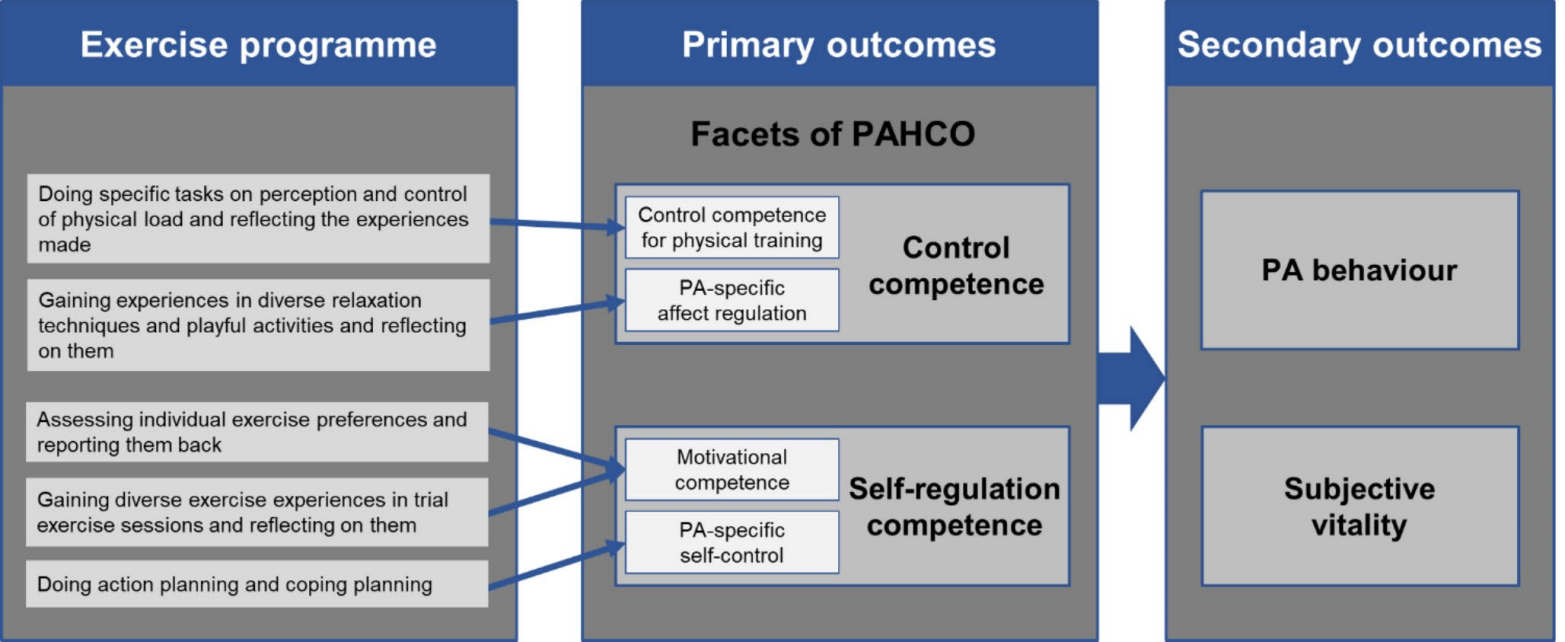
Elements of the multimodal exercise programme and associated outcomes *Notes.* PA = Physical activity; PAHCO = Physical activity-related health competence

Two of the PAHCO competences, control competence and self-regulation competence, are of particular importance in this study (see Fig. 1). *Control competence* describes an individual’s ability to gear their own activity to achieve positive effects in health and wellbeing. Two facets are differentiated here: (a) control competence for physical training allows people to model physical load in a health enhancing way, both by applying fitness knowledge (e.g., to choose longer but less intense bouts of exercise for continuous training) and by perceiving body signals (e.g., to use heart rate to control physical load in endurance training; Sudeck & Pfeifer, 2016); (b) PA-specific affect regulation helps people to plan and carry out activities in order to optimise benefits in wellbeing (e.g., to choose jogging in the forest to relax after a stressful day at work; Sudeck & Pfeifer, 2016). S*elf-regulation competence* refers to the motivation and volition that enables individuals to be regularly active. Here too, two facets are distinguished: (a) Motivational competence that allows people to choose a specific activity type that matches their preferences (e.g., to join a community-based hiking group because social contact is important for you; Schorno et al., 2021); (b) PA-specific self-control, which helps people to translate intentions into action by using behavioral change techniques, such as action planning (e.g., to plan when, where, how, and with whom you will be physically active in the future) and coping planning (e.g., to identify potential barriers in daily life and strategies to overcome them; see also Dishman & Ickes, 1981; Gollwitzer & Oettingen, 2016).

Various studies showed that the facets of PAHCO are not only related to the actual and habitual amount of PA, but also linked with health and wellbeing. This empirical evidence was found both in healthy adults (Carl, Grüne, et al., 2020; Schorno et al., 2021; Sudeck et al., 2018; Sudeck & Pfeifer, 2016) and in individuals with musculoskeletal, metabolic, cardiologic, oncologic, and chronic obstructive pulmonary diseases (Carl et al., 2021; Schmid et al., 2020; Sudeck & Pfeifer, 2016). However, the model has not yet been addressed in bariatric surgery patients even though it seems reasonable that its basic assumptions are also valid for this population.

To promote PAHCO, interventions should include not only a practical component (e.g., participating in PA) but also a theoretical one (e.g., teaching of knowledge about fitness). Furthermore, it is required to transfer these two components not in isolation but to combine them in a programme (Carl et al., 2020b; Haible et al., 2019). The instructional principle “reflection in or on action” is an effective method for combining practical and theoretical components (Baartman & Bruijn, 2011; Schön, 1983) but so far has received relatively little attention in the field of health promotion. One exception is the study by Volk et al. (2021), in which an intervention programme was designed and evaluated that particularly aimed to promote control competence for physical training in the context of physical education. Students performed specific activities (e.g., running with different intensities whilst monitoring their heart rate, creating a scatter plot diagram of heart rates during the activities), reflected, and discussed the experiences made together with other students and the teacher (e.g., group discussion about factors influencing heart rate; for more details see, Haible et al., 2019). Positive short-term effects of the described intervention on fitness knowledge and control competence for physical training were found (Volk et al., 2021). The instructional principle reflection on action was also applied in a study by Schorno et al. (2022), however they concentrated specificly on promoting the two facets of self-regulation competence in less active adults. Schorno et al. (2022) developed an exercise counselling approach that aims to help people find and regularly engage in a PA that suits them. Individuals gained diverse exercise experiences in trial sessions on-site (e.g., fitness activities vs. games vs. dance activities) and reflected on these experiences immediately after (e.g., discussion about which activity one liked and why). Afterwards, suitable exercise activities were deduced, and the counsellor assisted in developing a concrete action and coping plan. These two behaviour change techniques have already been found to be effective in adults with obesity (Göhner et al., 2012) and bariatric surgery patients (Bond et al., 2017). Positive short- and medium-term effects of the exercise counselling on motivational competence, PA-specific self-control, and self-reported exercise and sport volume were found (Schorno et al., 2022).

The aim of the present study was to examine the efficacy of a 3-month multimodal exercise programme focused on the promotion of PAHCO in a sample of bariatric surgery patients. The programme stands out by combining experiences with guided reflections (Schön, 1983; for more details see left box of Fig. 1), meaning that individuals do not only train, but they get more involved by discussing their exercise experiences. This study investigated (1) the impact of the programme on control competence for physical training, PA-specific affect regulation, motivational competence, and PA-specific self-control (primary outcomes; see Fig. 1). In accordance with previous findings (Göhner et al., 2012; Schorno et al., 2022; Volk et al., 2021), albeit partly in different populations, we expected positive treatment effects on all four facets of PAHCO mentioned above. Furthermore, this study examined (2) the impact on PA behaviour and subjective vitality as an indicator for wellbeing (secondary outcomes; see Fig. 1). Again, we expected positive treatment effects.

## Methods

### Study design

A single-centre RCT with two parallel groups with balanced randomisation (1:1) was performed (see CONSORT checklist in the supplementary material). Adult patients between 3 and 10 months after bariatric surgery were randomised to either the control group (CG) or intervention group (IG). While the CG received usual follow-up care alone, the IG additionally took part in a multimodal exercise programme for 3 months. After study completion, the CG received an individual exercise counselling, a free 10-pack subscription for a gym and all educational material used in the intervention as a compensation. Outcome assessments were performed pre intervention (t_1_), post intervention (t_2_), and at 3 months follow-up (t_3_) at the obesity outpatient clinic of the Bern University Hospital and at the Institute of Sport Science at University of Bern (see Fig. 1 in the supplementary material, for design and procedure details). Ethical approval was received from the Ethics Committee Bern (Reference: 202000900) and the trial was registered at clinicaltrials.gov (NCT04413812).

### Sample size calculation

Based on expected medium to large intervention effects (Göhner et al., 2012; Schorno et al., 2022; Volk et al., 2021), a sample size calculation was done using G*power (Faul et al., 2007). It indicated that a total of 34 participants are required to detect an effect size of f = 0.25 for the group x time interaction with a power of 80% at a two-sided significance level of 5%. In order to compensate for potential dropouts, targeted sample size was defined at 44 participants in total.

### Study population

Eligible participants were adult patients (≥ 18 years old) who had undergone standard Roux-en-Y gastric bypass or sleeve gastrectomy 3 to 10 months before start of the exercise programme, currently not engaging in structured exercise, and literate in German. Volunteers with contraindications to exercise engagement, a medical condition likely to interfere with the normal conduct of the study and interpretation of the study results, evidence of malnutrition, planned pregnancy, or abuse of illicit or prescription drugs were excluded.

### Recruitment, randomisation and masking

Patients were recruited from three local Centres of Excellence for Bariatric Surgery. Potential candidates were approached by phone and informed about the study. After provision of written informed consent, participants underwent a detailed eligibility screening and a baseline assessment of study outcomes. Thereafter, participants were randomised to the IG or CG. First, pairs of participants were created using non-bipartite matching according to age, sex, current BMI, and time since surgery. For each pair, one participant was randomly assigned to each group. The randomisation was performed using the software R (version 3.6.3) and the software package *nbpMatching*. The randomisation was performed by an independent researcher who had no other involvement in the study to ensure allocation concealment. Both the participant and the researcher became aware of the study group allocation upon completion of the baseline assessment.

### Study intervention and comparator

The intervention was a multimodal exercise programme consisting of 25 sessions over three months. Participants attended two sessions per week, one on-site at a fitness center or in the nearby outdoor areas (lasting 75 min/session) and one online (lasting 45 min/session). The on-site sessions were conducted in two groups of 10 participants each. The online sessions consisted of either physical group training, educational workshop, or individual counselling. All online sessions except the individual counselling were recorded and uploaded on an online platform to allow for a later use in case somebody could not be present at the given time. Each participant received a course book containing organisational information, the worksheets used in the sessions, and a set of strength exercises. In the on-site and online group trainings, exercises were individually adapted to the fitness level of the participants. All sessions were led by three trained students in Sport and Movement Science and medically supervised.

Figure 1 shows the content of the exercise programme and its relationship to the control and self-regulation competences, including their facets and the specification of primary and secondary outcomes. Table 1 in the supplementary material gives an additional detailed overview. Most of the sessions were instructed using the principles of reflection in action (i.e., specific task to perceive body signals while running in different intensities) or reflection on action (i.e., reflecting on perceived body signals from previous activities), which both have the potential to promote an individual’s competence (Schön, 1983). Sessions addressing control competence for physical training were based on the intervention programme of Volk et al. (2021). Sessions addressing motivational competence were inspired by the counselling concept of Schorno et al. (2022) and sessions addressing PA-specific self-control were based on the intervention programme of Göhner et al. (2012).

The comparator consisted of standard post-bariatric surgery follow-up care alone, involving medical appointments with a bariatric physician and a dietitian at 3 months, 6 months, 9 months, and 12 months post-operatively.

### Measures for the primary and secondary outcomes

Primary outcomes of the trial were the four mentioned facets of PAHCO. Control competence for physical training (6 items), PA-specific affect regulation (4 items), and PA-specific self-control (3 items) were measured using scales validated by Sudeck and Pfeifer (2016). In contrast, motivational competence (4 items) was assessed with a scale validated by Schorno et al. (2021). Table 2 in the supplementary material gives more details about the scales used, including reliabilities and example item wording.

The secondary outcomes of the trial were PA behaviour and subjective vitality. PA behaviour was assessed via self-report using the Physical Activity, Exercise, and Sport Questionnaire (Fuchs et al., 2015). Based on the information provided by the questionnaire, a weekly volume of exercise in minutes was calculated. Additionally, PA behaviour was assessed via accelerometers. The three-axial acceleration sensors, Move 4 (movisens GmbH, Karlsruhe, Germany), were used with a measurement range of ± 8 g and a sampling rate of 64 Hz. Participants were instructed to attach the Move 4 to the right side of their hip for seven consecutive days during waking periods. Move 4 data were downloaded and analysed using Movisens DataAnalyzer. Measurements were considered valid if they contained at least data over 4 days with a minimal wear time of 8 h per day (Burchartz et al., 2020). Weekly volume of moderate to vigorous PA in minutes was computed based on all valid days per person. To assess subjective vitality (6 items), a validated German version of the vitality scale was used (Goldbeck et al., 2019).

The aforementioned primary and secondary outcomes were recorded at all three measurement time points (t_1_-t_3_). One exception was the accelerometer-based moderate to vigorous PA, which, for pragmatic reasons, was only assessed pre intervention (t_1_) and at 3 months follow-up (t_3_; see Fig. 1 in the supplementary material).

### Statistical analysis

Statistical analysis was performed using R version 4.0.2 (The R Foundation for Statistical Computing, Vienna, Austria). Analyses for primary and secondary outcomes were conducted using Generalized Estimation Equations (GEE) with the main effects of time (time entered as a factor, t_1_-t_2_, t_1_-t_3_), group and the group-time interactions as explanatory variables. These models were selected in order to adjust for potential baseline differences. For all models, an independent correlation structure, assuming a Gaussian error distribution, was used. No imputation of missing data was performed since GEEs are specifically designed to accommodate missing values. For all analyses, *p* < 0.05 was considered statistically significant. Models were implemented using the R package “geepack” (Halekoh et al., 2006). The standardised regression coefficients (β_StdY_ = *b*_unstandardised_/*SD*[Y]) were calculated to describe the size of the treatment effect. A β_StdY_ = 0.20–0.49 was interpreted as small, a β_StdY_ = 0.50–0.79 as medium, and a β_StdY_ ≥ 0.80 as large effects (Cohen, 1988).

## Results

### Participant characteristics

**Fig. 2:**
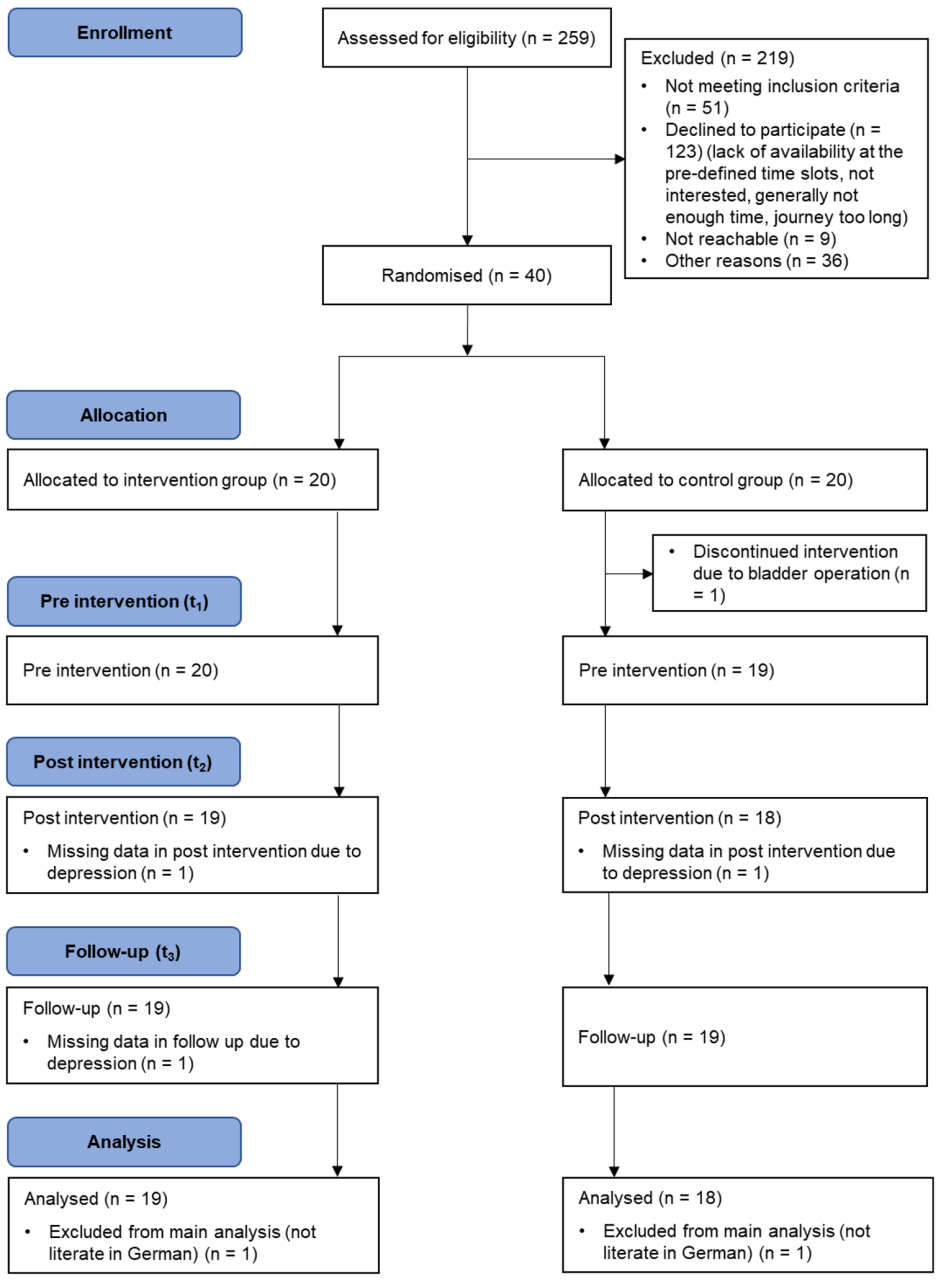
Flow diagram of participants

Of 259 patients initially approached, 250 responded and 91 expressed interest and were screened for trial eligibility. A total of 40 patients were considered eligible for participation, had time availability at the pre-dimed slots, and consented to randomisation (see Fig. 2). One person dropped out of the study pre intervention (t_1_) due to a bladder operation. In addition, two participants had to be excluded from the final analysis due to insufficient skills in German language, which became noticeable after the beginning of the programme. Consequently, the analysed sample consisted of 19 individuals from the IG and 18 individuals from the CG.

Despite having access to a relatively large number of potential study participants, we did not reach the recruitment target of n = 44 during the 3-month study recruitment phase (recruitment rate: 16.26%). The main reason for non-participation was lack of time availabilities, particularly during the pre-defined slots (for more details see Fig. 2).

Mean time since surgery was 5.3 months. Participants were 62% women, had a mean age of 43.95 years (*SD* = 10.36), and a mean pre-surgery weight of 122.99 kg (*SD* = 22.93). Their mean BMI was 41.88 kg/m^2^ (*SD* = 6.20). 51% of the participants had a gastric bypass surgery and 49% a sleeve gastrectomy. Of 25 possible exercise sessions, those randomised to the IG attended a mean of 16.8 sessions (range: 9–22 sessions). No serious adverse events occurred during the trial. Table 3 in the supplementary material presents all baseline characteristics and Table 4 in the supplementary material shows the descriptives of the study outcomes. Overall, there were 16 missing values, which correspond to 3.09% (t_2_: twelve missings in the PAHCO facets; t_3_: four missings in the PAHCO facets).

### Intervention effects on primary outcomes

Analyses revealed significant group-time interactions for the PAHCO facet control competence for physical training, both between t_1-_t_2_ and between t_1_-t_3_. Compared to the members of the CG, those of the IG were able to significantly increase their competence not only by the end of the exercise programme (*p* = 0.011), but also at 3 months of follow-up (*p* < 0.001; for more details see Table 1). Effect sizes are classified as medium and large respectively (t_1_-t_2_: β_StdY_ = 0.71; t_1_-t_3_: β_StdY_ = 0.87). However, there was no significant group by time interaction effect for PA-specific affect regulation (t_1_-t_2_: *p* = 0.600, β_StdY_ = 0.16; t_1_-t_3_: *p* = 0.110, β_StdY_ = 0.41).

**Table 1:** Effects of the Generalized Estimation Equations – primary outcomes

Effects		*b*	SE	β_StdY_	*p*	95% Confidence Intervals	
						Lower	Upper
*Control competence for physical training (t*_*1*_ *-t*_*2*_ * and t*_*1*_ *-t*_*3*_ *)*							
	Intercept	3.06	0.20	-0.47	**< 0.001**	2.67	3.44
	Time t_1_-t_2_	0.42	0.14	0.48	**0.003**	0.14	0.69
	Time t_1_-t_3_	0.32	0.16	0.38	**0.037**	0.02	0.63
	Group	-0.12	0.26	-0.14	0.650	-0.64	0.40
	Time t_1_-t_2_ × group	0.61	0.24	0.71	**0.011**	0.14	1.08
	Time t_1_-t_3_ × group	0.75	0.20	0.87	**< 0.001**	0.35	1.15
*PA-specific affect regulation (t*_*1*_ *-t*_*2*_ * and t*_*1*_ *-t*_*3*_ *)*							
	Intercept	2.78	0.24	-0.43	**< 0.001**	2.30	3.25
	Time t_1_-t_2_	0.35	0.19	0.34	0.059	-0.01	0.72
	Time t_1_-t_3_	0.29	0.15	0.28	0.050	0.00	0.58
	Group	0.26	0.37	0.26	0.480	-0.46	0.99
	Time t_1_-t_2_ × group	0.16	0.31	0.16	0.600	-0.44	0.76
	Time t_1_-t_3_ × group	0.42	0.26	0.41	0.110	-0.09	0.92
*Motivational competence (t*_*1*_ *-t*_*2*_ * and t*_*1*_ *-t*_*3*_ *)*							
	Intercept	3.36	0.23	-0.52	**< 0.001**	2.91	3.81
	Time t_1_-t_2_	0.34	0.15	0.41	**0.020**	0.05	0.64
	Time t_1_-t_3_	0.32	0.19	0.38	0.095	-0.06	0.69
	Group	0.12	0.29	0.15	0.670	-0.45	0.70
	Time t_1_-t_2_ × group	0.46	0.25	0.57	0.052	-0.00	0.95
	Time t_1_-t_3_ × group	0.44	0.25	0.55	0.059	-0.04	0.93
*PA-specific self-control (t*_*1*_ *-t*_*2*_ * and t*_*1*_ *-t*_*3*_ *)*							
	Intercept	3.06	0.28	-0.26	**< 0.001**	2.67	3.44
	Time t_1_-t_2_	0.20	0.17	0.21	0.240	0.14	0.69
	Time t_1_-t_3_	0.26	0.22	0.27	0.250	0.02	0.63
	Group	-0.11	0.35	-0.12	0.750	-0.64	0.40
	Time t_1_-t_2_ × group	0.54	0.27	0.57	**0.048**	0.00	1.08
	Time t_1_-t_3_ × group	0.35	0.31	0.37	0.260	-0.26	0.97

*Note*. PA = Physical activity; *p* values < 0.05 noted in bold. The control group and baseline (t_1_) served as reference levels for the regression model

In terms of motivational competence, results showed a marginally non-significant group by time interaction in favour of the IG, both between t_1_-t_2_ (*p* = 0.052) and between t_1_ and t_3_ (*p* = 0.059). Both effects are classified as medium (t_1_-t_2_: β_StdY_ = 0.57; t_1_-t_3_: β_StdY_ = 0.55). Analyses also revealed a significant and medium group by time interaction for PA-specific self-control between t_1_-t_2_ (*p* = 0.048; β_StdY_ = 0.57), suggesting that members of the IG were able to increase their PA-specific control-competence after the exercise programme compared to the CG. In contrast, no significant group by time interaction effect was observed between t_1_-t_3_ (*p* = 0.260, β_StdY_ = 0.37).

### Intervention effects on secondary outcomes

Regarding the self-reported exercise behaviour, results revealed significant group by time interaction effects, both between t_1_-t_2_ (*p* < 0.001) and between t_1_-t_3_ (*p* = 0.007) in favour of the IG (for more details see Table 2). More specifically, the IG’s average self-reported amount of exercise increased from 87 min/week to 154 min/week at t_2_ and then dropped again slightly to 115 min/week at t_3_ (see Table 4 in supplementary material). In contrast, the CG’s average decreased gradually from 105 min/week at t_1_ to 55 min/week and 24 min/week at the two consecutive measurement points. Both group by time interaction effects can be classified as large (t_1_-t_2_: β_StdY_ = 1.44; t_1_-t_3_: β_StdY_ = 1.01). In contrast to the self-reported measure, no group by time interaction effect was found for device-based measure of PA behaviour (*p* = 0.33; β_StdY_ = -0.28).

**Table 2:** Effects of the Generalized Estimation Equations – secondary outcomes

Effects		*b*	SE	β_StdY_	*p*	95% Confidence Intervals	
						Lower	Upper
*PA behaviour: self-reported exercise (logarithmised; min/week; t*_*1*_ *-t*_*2*_ * and t*_*1*_ *-t*_*3*_ *)*							
	Intercept	1.23	0.25	0.08	**< 0.001**	0.75	1.71
	Time t_1_-t_2_	-0.48	0.20	-0.19	**0.019**	-0.88	-0.08
	Time t_1_-t_3_	-0.78	0.26	-0.24	**0.002**	-1.28	-0.28
	Group	-0.20	0.36	-0.19	0.568	-0.90	0.49
	Time t_1_-t_2_ × group	1.57	0.30	1.44	**< 0.001**	0.98	2.16
	Time t_1_-t_3_ × group	1.10	0.40	1.01	**0.007**	0.31	1.89
*PA behaviour: device-based moderate to vigorous PA (min/week; t*_*1*_ *-t*_*3*_ *)*							
	Intercept	295.59	57.25	0.17	**< 0.001**	183.39	407.79
	Time t_1_-t_3_	-25.17	35.55	-0.12	0.480	-94.86	44.51
	Group	-17.54	74.42	-0.08	0.810	-163.40	128.32
	Time t_1_-t_3_ × group	-61.45	63.36	-0.28	0.330	-195.64	62.74
*Subjective vitality (t*_*1*_ *-t*_*2*_ * and t*_*1*_ *-t*_*3*_ *)*							
	Intercept	4.68	0.32	-0.22	**< 0.001**	4.05	5.31
	Time t_1_-t_2_	0.20	0.29	0.15	0.480	-0.36	0.77
	Time t_1_-t_3_	-0.04	0.28	-0.03	0.872	-0.585	0.50
	Group	-0.07	0.46	-0.05	0.885	-0.98	0.84
	Time t_1_-t_2_ × group	0.57	0.44	0.45	0.192	-0.29	1.43
	Time t_1_-t_3_ × group	1.03	0.36	0.81	**0.005**	0.32	1.75

*Note*. PA = Physical activity; *p* values < 0.05 noted in bold. The control group and baseline (t_1_) served as reference levels for the regression model

Regarding subjective vitality, a significant group by time interaction effect was found in favour of the IG, but only between t_1_-t_3_ (*p* = 0.005), though not between t_1_-t_2_ (*p* = 0.192). This effect size can be classified as large (t_1_-t_3_: β_StdY_ = 0.81).

## Discussion

In this work, we designed and evaluated a novel 3-month multimodal exercise programme focussing on specific facets of PAHCO in patients who recently underwent bariatric surgery. We found that the multimodal exercise programme was successful in enhancing control competence for physical training and PA-specific self-control and positively influenced self-reported exercise and subjective vitality. Conversely, we found no statistically significant positive effects on PA-specific affect regulation, motivational competence, and device-based PA.

The observed positive impact on participants’ control competence for physical training can be considered as large and in line with a previous intervention study (Volk et al., 2021). Of note, the effects were not only short-term, but persisted until the follow-up assessment three months after completion of the programme. This means that the intervention empowered participants to apply training-specific knowledge and effectively select and structure activities for promoting physical health in daily life. The present research revealed larger treatment effects than in a recent study published by Volk et al. (2021). This could be due to differences in the study populations such as age (adolescents vs. adults) and/or biopsychosocial health status (healthy individuals vs. bariatric surgery individuals). It is possible that adults with complex biopsychosocial needs, such as the population under investigation, particularly benefit from such a programme as they appreciate its personal utility and relevance and therefore are more willing to improve their PAHCO than healthy adolescents in compulsory education. Overall, the observed success in our study population suggests that the PAHCO model with the intervention sessions developed by Haible et al. (2019) are well applicable to complex clinical settings and patients groups such as those after bariatric surgery.

A possible explanation for the lack of a positive treatment effect on the PA-specific affect regulation may be that it was not sufficiently addressed by the exercise programme (see Table 1 in the supplementary material). It is important to note that promoting affect regulation, or in other words, the competence to steer PA in order to optimise psychological wellbeing, remains an under-researched area. Future research should focus on which personal, environmental, and activity-related factors influence intra-individual differences in affective response to PA (e.g., valence, arousal; see Bourke et al., 2020) thereby setting the ground for more effective intervention.

Although the present study revealed medium effects of the exercise programme on motivational competence, they remained non-significant, probably influenced by our rather small sample size. However, compared to a previous study with less active, healthy adults (Schorno et al., 2022), this research revealed slightly smaller treatment effects. One can speculate that this is related to differences in characteristics in the study population or the delivery mode of the individual exercise counselling. In the study by Schorno et al. (2022) the trial exercise sessions and the reflection about the experiences made took place on the same day. In contrast, the trial exercise sessions and the reflection in the present study were spread over several days for logistic reasons. It is therefore possible, that lack of interweaving between practical experience and reflection in terms of time and space diminished the impact on individual learning (Schön, 1983).

With regard to PA-specific self-control, this study showed a positive, medium treatment effect at the end of the exercise programme. This means that participants felt more competent in implementing regular PA in daily life. This result is in line with prior studies with less active but healthy adults (Schorno et al., 2022), individuals with obesity (Göhner et al., 2012), and bariatric surgery patients (Bond et al., 2016), which also revealed medium to high treatment effects on related volitional outcomes. However, the present findings at the follow-up assessment three months after completion of the programme looked somewhat different. Although the participants of the exercise programme maintained their increased level of PA-specific self-control on average at t_3_, the participants of the CG were also able to improve their competence between t_2_ and t_3_, resulting in a not significant treatment effect at follow-up. Consequently, a question warranting further research is, whether the exercise programme needs to be prolonged or supplemented with a booster session after its completion to maximize its benefits in the long run.

A secondary outcome of this trial was self-reported exercise behaviour. The exercise programme resulted in increased self-reported exercise for members of the IC compared to those of the CG. In absolute terms, members of the IG were able to raise their exercise level by 28 min/week. This is an increase that has shown to be associated with positive health effects (e.g., a reduction mortality; see Wen et al., 2011). However, this large effect on the self-reported exercise volume found in our study need to be interpreted with caution, as no treatment effect was found on device-based moderate to vigorous PA. Our results are consistent with the few existing RCT, which also found no effects of exercise programmes on accelerometer-based PA in bariatric surgery patients (Bellicha et al., 2018). The fact that self-reports differ from device-based assessed PA has been observed and discussed in other studies with adults who underwent bariatric surgery (Herring et al., 2017). Discrepancies may be explained by several reasons: Firstly, the two measures have different scopes. Whereas self-reported data reflect structured exercise activities performed during leisure time, accelerometer-based data reflect moderate to vigorous PA, including structured and unstructured PA, not only performed during leisure time, but also at work or for transportation (Burchartz et al., 2020; Fuchs et al., 2015; Strath et al., 2013). Secondly, a potential underestimation of acelerometer-based PA in the IG. Despite technical advances in recent years, accelerometers are not (yet) able to measure all physical activities. For instance, cycling, a session on a cross trainer, or strength training can not be adequately captured with a device (Burchartz et al., 2020). A closer look at the data shows that the above activities were frequently reported specifically by patients of the IG at t_2_ and t_3_. Thirdly, an overestimation of self-reported exercise in the IG. Due to social desirability, patients of the IG might have reported more or longer exercise activities after they participated in the programme (Nigg et al., 2020).

A large effect was found for the multimodal exercise programme on subjective vitality at follow-up, meaning that members of the IG felt more energised and vital compared to the CG. This result is in line with findings of previous observational studies (Carl, Grüne, et al., 2020; Schmid et al., 2020). The positive finding on subjective vitality supports the idea that exercise programme should also focus on qualitative aspects of PA and not simply quantitative aspects and teaching the formula “the more, the better” (Sudeck & Pfeifer, 2016).

The strengths of the present RCT include its rigorous design and the novel application of the PAHCO model in the bariatric surgery population. However, we acknowledge some limitations. Firstly, participants included mostly had a rather high PA level at baseline (see Table 4 in supplementary material), which may limit the generalisability of the present findings to all bariatric surgery patients. Secondly, we did not examine the effect of the exercise programme on movement competence, which is also integrated in the PAHCO model alongside control competence and self-regulation competence (Sudeck & Pfeifer, 2016). Movement competence allows people to participate in diverse leisure activities and to master motor challenges of daily life (e.g., motor abilities and motor skills for riding a bike or gymnastics) and is therefore an important competence worth to consider in further research. Thirdly, our study design does not allow a concrete statement about which programme element has which effect on PAHCO, PA behavior, and subjective vitality, as we evaluated the exercise programme as a whole. Finally, the sample size of the study can be considered to be limited, resulting in low power to detect small effects.

## Conclusion

Findings from this RCT overall revealed that a multimodal exercise programme was effective in improving single facets of PAHCO, self-reported exercise, and wellbeing among bariatric surgery patients. This study therefore provides a foundation for future research for the use of the PAHCO model (Sudeck & Pfeifer, 2016) to optimise long-term post bariatric surgery outcomes. In particular, larger scale RCTs and longer follow-up periods to determine maintenance of outcomes from such a novel programme are needed. Additionally, optimal timing, frequency, and feasibility of implementation in routine care need to be defined.

### Electronic supplementary material

Below is the link to the electronic supplementary material.

Supplementary Material 1

### Electronic supplementary material

Below is the link to the electronic supplementary material.

Supplementary Material 2

### Electronic supplementary material

Below is the link to the electronic supplementary material.

Supplementary Material 3

### Electronic supplementary material

Below is the link to the electronic supplementary material.

Supplementary Material 4

### Electronic supplementary material

Below is the link to the electronic supplementary material.

Supplementary Material 5

## Data Availability

The datasets generated during and/or analysed during the current study are available from the corresponding author on reasonable request.
