# Supplementary material for: Fostering physical activity-related health competence after bariatric surgery with a multimodal exercise programme: A randomised controlled trial
Source: J Behav Med. 2023 Mar 2;46(5):709–19. doi: 10.1007/s10865-023-00398-7 (PMC10558379; doi:10.1007/s10865-023-00398-7)
Supplement: Supplementary file 1 — Supplementary Material 1 [file 10865_2023_398_MOESM1_ESM.docx]

**
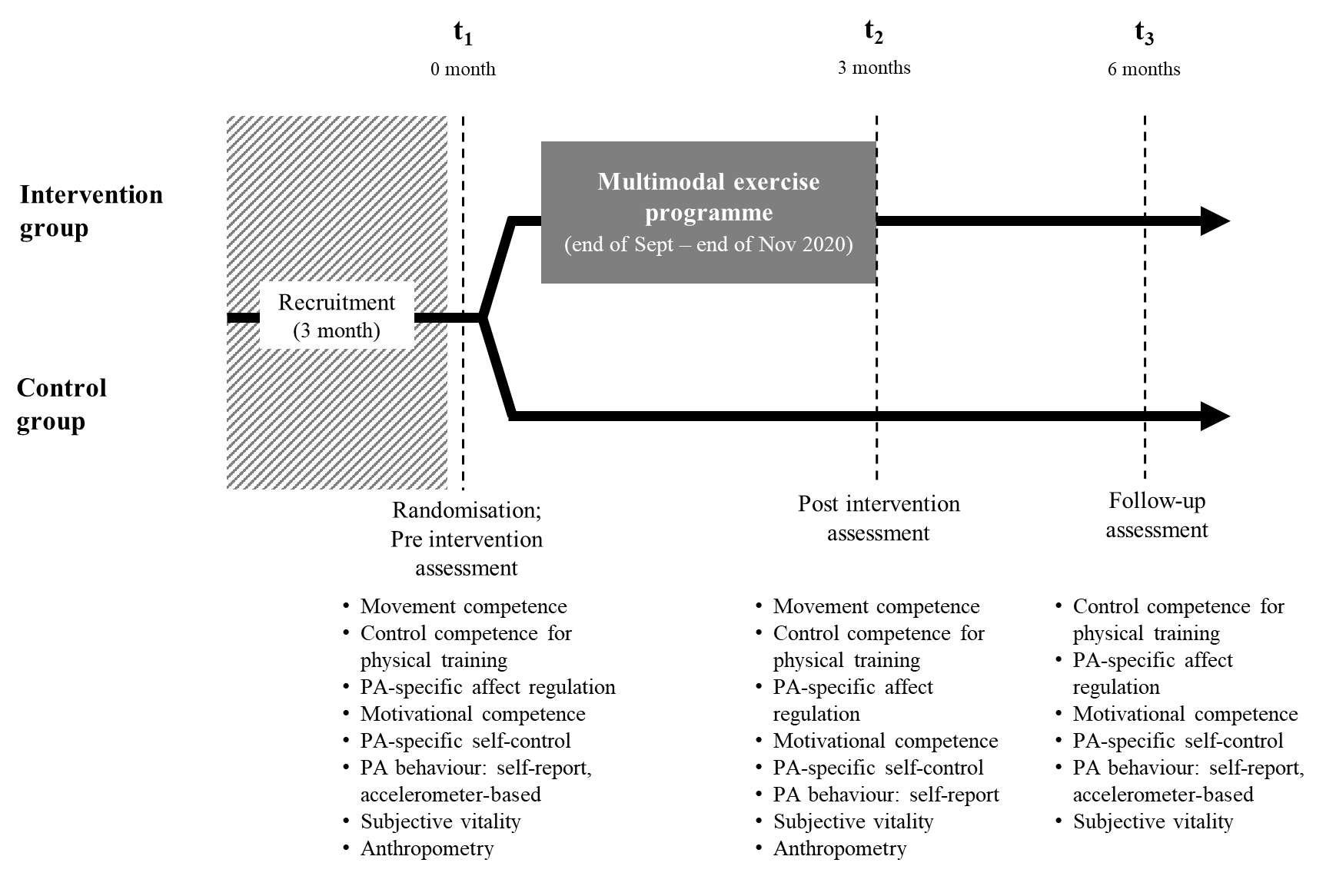
**

**Electronic supplementary material,** **Figure 1.** Study design, procedures, assessments and measures.

*Notes.* PA = Physical activity.
