# Supplementary material for: Fostering physical activity-related health competence after bariatric surgery with a multimodal exercise programme: A randomised controlled trial
Source: J Behav Med. 2023 Mar 2;46(5):709–19. doi: 10.1007/s10865-023-00398-7 (PMC10558379; doi:10.1007/s10865-023-00398-7)
Supplement: Supplementary file 2 — Supplementary Material 2 [file 10865_2023_398_MOESM2_ESM.docx]

**Electronic supplementary material,** **Table 1.** Organisation, topics and focused outcome of the 3-month exercise programme

| **♯** | **Organisation** | **Topic** | **Source** | **Focused PAHCO facet** |
| --- | --- | --- | --- | --- |
| 1 | On-site group training | Kick-off event: get to know each other | - | - |
| 2 | Online group workshop | Effects of PA on health and wellbeing, basic knowledge of physical training | - | Control competence for physical training |
| 3 | On-site group training | Perception of physiological responses to PA | Lesson 1 by Haible et al. (2019) | Control competence for physical training |
| 4 | Online group training |  |  |  |
| 5 | On-site group training | Perception and measurement of heart rate | Lesson 2 by Haible et al. (2019) | Control competence for physical training |
| 6 | Online group training |  |  |  |
| 7 | On-site group training | Training in a playful way: small games | - | PA-specific affect regulation |
| 8 | Online group workshop | Overcome PA barriers in everyday life: Learning the behaviour change technique coping planning^1^ | Göhner et al. (2012) | PA-specific self-control |
| 9 | On-site group training | Perception and measure of perceived exertion | Lesson 3 by Haible et al. (2019) | Control competence for physical training |
| 10 | Online group training |  |  |  |
| 11 | On-site group training | Introduction to strength training with machines I | - | Control competence for physical training |
| 12 | Online group training | Introduction to strength training with own bodyweight | - | Control competence for physical training |
| 13 | On-site group training | Introduction to strength training with machines II | - | Control competence for physical training |
| 14 | Online group training | Strength training with own bodyweight | - | Control competence for physical training |
| 15 | On-site group training | Trial exercise session: Dance and Taekwondo | Schmid et al. (2020); Schorno et al. (2022) | Motivational competence |
| 16 | Online group training | Trial exercise session: Yoga |  |  |
| 17 | On-site group training | Trial exercise session: Aquafit |  |  |
| 18 | Individual counselling | Find an activity that suits one’s own preferences: Assessing individual preferences, reporting them back, reflecting on the experiences in the exercise sessions and discussing about suitable activities |  |  |
| 19 | On-site group training | Control of physical load: endurance training | Lesson 5 by Haible et al. (2019) | Control competence for physical training |
| 20 | Online group training | Strength and endurance circuit, progressive muscle relaxation | - | Control competence for physical training, PA-specific affect regulation |
| 21 | On-site group training | Control of physical load: strength training with own bodyweight | Lesson 4 by Haible et al. (2019) | Control competence for physical training |
| 22 | Online group training | Strength training with own bodyweight, autogenic training | - | PA-specific affect regulation |
| 23 | On-site group training | Training in a playful way: small games | - | PA-specific affect regulation |
| 24 | Online group workshop | Implement a suitable activity in everyday life: Learning the behaviour change technique action planning^1^ | Göhner et al. (2012) | PA-specific self-control |
| 25 | On-site group training | Closing event: repetition and quiz of inputs | - | Control competence for physical training |

*Note.* ^1^ Based on Mitchie et al. (2013) taxonomy of behaviour change techniques, coping planning is coded as 1.2 and action planning is coded as 1.4.
