# Supplementary material for: Fostering physical activity-related health competence after bariatric surgery with a multimodal exercise programme: A randomised controlled trial
Source: J Behav Med. 2023 Mar 2;46(5):709–19. doi: 10.1007/s10865-023-00398-7 (PMC10558379; doi:10.1007/s10865-023-00398-7)
Supplement: Supplementary file 3 — Supplementary Material 3 [file 10865_2023_398_MOESM3_ESM.docx]

**Electronic supplementary material,** **Table 2.** Overview of the scales used

|  | | **Cronbach’s α** | | | **Measure-ment points** | **Number of items** | **Response format** | **Exemplary items** |
| --- | --- | --- | --- | --- | --- | --- | --- | --- |
|  | | t_1_ | t_2_ | t_3_ |  |  |  |  |
| Primary outcome: PAHCO | | |  |  | |  |  |  |
|  | Control competence for physical training | .77 | .88 | .88 | t_1_, t_2_, t_3_ | 6 | 1 (“disagree completely”) to 5 (“agree completely”) | “If I want to enhance my health by strengthening trunk muscles (back, stomach), I am confident that I know the right exercises to do so” or “I can use my body signals (pulse, breathing speed) very well to gauge and regulate the amount of physical load” |
|  | PA-specific affect regulation | .96 | .89 | .90 |  | 4 |  | “I am very able to improve my depressed mood by exercising” or “If I am feeling down, I can distract myself well through physical activity” |
|  | Motivational competence | .85 | .86 | .78 |  | 4 |  | “I know exactly what is important for me in an exercise and sport activity so that I like it” or “I find it very easy to assess what characterises different exercise and sport activities” |
|  | PA-specific self-control | .88 | .75 | .85 |  | 3 |  | “I stick with my plan to exercise and am not easily distracted from that plan” or “When I decide to exercise more, I am very disciplined in implementing this plan” |
| Secondary outcomes: PA behaviour and subjective vitality | | | | | |  |  |  |
|  | Self-reported exercise (min/week) | - | | | t_1_, t_2_, t_3_ | - | - | - |
|  | Accelerometer-based moderate to vigorous PA (min/week) | - | | | t_1_, t_3_ | - | - | - |
|  | Subjective vitality | .93 | .92 | .91 | t_1_, t_2_, t_3_ | 6 | 1 (“not at all true”) to 7 (“very true”) | “I feel energised” or “I feel alive and vital” |

*Notes.* PA = Physical activity; PAHCO = Physical activity-related health competence.
