# Supplementary material for: Fostering physical activity-related health competence after bariatric surgery with a multimodal exercise programme: A randomised controlled trial
Source: J Behav Med. 2023 Mar 2;46(5):709–19. doi: 10.1007/s10865-023-00398-7 (PMC10558379; doi:10.1007/s10865-023-00398-7)
Supplement: Supplementary file 4 — Supplementary Material 4 [file 10865_2023_398_MOESM4_ESM.docx]

**Electronic supplementary material,** **Table 3.** Characteristics of the study population (mean ± *SD*, or %)

|  | | **Intervention group**  *n* = 19 | **Control group**  *n* = 18 |
| --- | --- | --- | --- |
| *Sociodemographics and anthropometry* | | | |
|  | Age (years), range 23-69 | 44.5 ± 9.8 | 43.3 ± 11.0 |
|  | Gender (male:female) | 7 (37%):12 (63%) | 7 (38.9%):11 (61.1%) |
|  | BMI (kg/m^2^), pre-surgery | 41.9 ± 6.5 | 41.9 ± 6.1 |
|  | BMI (kg/m^2^) at enrollment | 34.0 ± 5.1 | 33.1 ± 5.1 |
|  | Time since surgery (months) | 5.3 ± 3.1 | 5.2 ± 2.8 |
| *Surgery type* | | | |
|  | Sleeve gastrectomy | 12 (63.8%) | 6 (33.3%) |
|  | Roux-en-Y-Magenbypass | 7 (36.8%) | 12 (66.7%) |
| *Level of education* | | | |
|  | First-level education  (e.g., primary school) | 3 (15.8%) | 3 (16.7%) |
|  | Second-level education  (e.g., apprenticeship) | 11 (57.9%) | 11 (61.1%) |
|  | Third-level education  (e.g., university) | 3 (15.8%) | 2 (11.1%) |
|  | N.A. | 2 (10.5%) | 2 (11.1%) |
| *Smoking* | | | |
|  | Yes | 5 (26.3%) | 4 (22.2%) |
|  | Former smoker | 4 (21.1%) | 4 (22.2%) |
|  | No | 8 (42.1%) | 9 (50.0%) |
|  | N.A. | 2 (10.5%) | 1 (5.6%) |
| *Nationality* | | | |
|  | Swiss | 15 (79.0%) | 15 (83.3%) |
|  | Others | 2 (10.5%) | 2 (11.1%) |
|  | N.A. | 2 (10.5%) | 1 (5.6%) |

*Note.* N. A. = data not available.
