# Supplementary material for: Fostering physical activity-related health competence after bariatric surgery with a multimodal exercise programme: A randomised controlled trial
Source: J Behav Med. 2023 Mar 2;46(5):709–19. doi: 10.1007/s10865-023-00398-7 (PMC10558379; doi:10.1007/s10865-023-00398-7)
Supplement: Supplementary file 5 — Supplementary Material 5 [file 10865_2023_398_MOESM5_ESM.docx]

**Electronic supplementary material,** **Table 4.** Descriptives of the primary and secondary outcomes (mean ± *SD*)

|  | | **Intervention group** | **Control**  **group** |
| --- | --- | --- | --- |
| *Control competence [1-5]* | | | |
|  | Control competence for physical training t_1_ | 2.92 ± 0.76 | 3.06 ± 0.85 |
|  | Control competence for physical training t_2_ | 3.96 ± 0.63 | 3.47 ± 0.93 |
|  | Control competence for physical training t_3_ | 4.00 ± 0.53 | 3.38 ± 0.87 |
|  | PA-specific affect regulation t_1_ | 2.99 ± 1.22 | 2.78 ± 1.05 |
|  | PA-specific affect regulation t_2_ | 3.56 ± 0.69 | 3.13 ± 1.05 |
|  | PA-specific affect regulation t_3_ | 3.75 ± 0.82 | 3.07 ± 1.03 |
| *Self-regulation competence [1-5]* | | | |
|  | Motivational competence t_1_ | 3.47 ± 0.77 | 3.36 ± 1.00 |
|  | Motivational competence t_2_ | 4.29 ± 0.57 | 3.72 ± 0.94 |
|  | Motivational competence t_3_ | 4.25 ± 0.48 | 3.68 ± 0.70 |
|  | PA-specific self-control t_1_ | 2.91 ± 0.93 | 3.06 ± 1.20 |
|  | PA-specific self-control t_2_ | 3.69 ± 0.82 | 3.29 ± 0.81 |
|  | PA-specific self-control t_3_ | 3.56 ± 1.01 | 3.31 ± 0.76 |
| *PA behaviour (min/week)* | | | |
|  | Self-reported exercise t_1_ | 86.87 ± 122.77 | 104.50 ± 164.13 |
|  | Self-reported exercise t_2_ | 154.33 ± 101.75 | 55.28 ± 102.92 |
|  | Self-reported exercise t_3_ | 115.13 ± 140.47 | 24.21 ± 53.03 |
|  | Accelerometer-based moderate to vigorous PA t_1_ | 266.8 ± 213.30 | 295.3 ± 242.90 |
|  | Accelerometer-based moderate to vigorous PA t_3_ | 184.2 ± 160.10 | 266.9 ± 240.50 |
| *Subjective vitality [1-7]* | | | |
|  | Subjective vitality t_1_ | 4.62 ± 1.50 | 4.68 ± 1.40 |
|  | Subjective vitality t_2_ | 5.39 ± 1.15 | 4.88 ± 1.25 |
|  | Subjective vitality t_3_ | 5.60 ± 0.92 | 4.63 ± 1.16 |

*Notes.* PA = physical activity.
